# Differential significance of molecular subtypes which were classified into *EGFR* exon 19 deletion on the first line afatinib monotherapy

**DOI:** 10.1186/s12885-020-6593-1

**Published:** 2020-02-06

**Authors:** Nahomi Tokudome, Yasuhiro Koh, Hiroaki Akamatsu, Daichi Fujimoto, Isamu Okamoto, Kazuhiko Nakagawa, Toyoaki Hida, Fumio Imamura, Satoshi Morita, Nobuyuki Yamamoto

**Affiliations:** 1 Internal Medicine III, Wakayama Medical University, 811-1 Kimiidera, Wakayama, 641-8509 Japan; 2 Department of Respiratory Medicine, Kobe City Medical Center General Hospital, Kobe, Japan; 3 Research Institute for Diseases of the Chest, Graduate School of Medical Sciences, Kyushu University, Fukuoka, Japan; 4 Department of Medical Oncology, Faculty of Medicine, Kindai University, Osaka, Japan; 5 Department of Thoracic Oncology, Aichi Cancer Center, Nagoya, Japan; 6 Department of Thoracic Oncology, Osaka International Cancer Institute, Osaka, Japan; 7 Department of Biomedical Statistics and Bioinformatics, Kyoto University Graduate School of Medicine, Kyoto, Japan

**Keywords:** NSCLC, Afatinib, *EGFR* mutation, Exon 19 deletion, Molecular subtypes

## Abstract

**Background:**

Epidermal growth factor receptor (*EGFR)-*sensitizing mutation, exon 19 deletion consists of several molecular variants. Influences of these variants on clinical response to EGFR tyrosine kinase inhibitors remain elusive.

**Methods:**

West Japan Oncology Group 8114LTR is a prospective, multi-institutional biomarker study. Treatment naïve, advanced non-small-cell lung cancer patients with *EGFR*-sensitizing mutation received afatinib monotherapy. We conducted a preplanned subset analysis of patients harboring exon 19 deletion. Tumor tissue exon 19 deletion molecular variants were identified by blocking-oligo-dependent polymerase chain reaction (PCR) and by Luminex Technology. Plasma cfDNA was also obtained before and after the treatment and *EGFR* mutations were detected with multiplexed, pico-droplet digital PCR assay.

**Results:**

Among 57 registered patients, twenty-nine patients were exon 19 deletion. Tissue DNA and cfDNA were available in 26 patients. Among the detected seven molecular variants, the most frequent was p.E746_A750delELREA (65.4%). According to the various classifications of molecular variants, twenty one (80.8%) were classified into 15-nucleotide deletion, one (3.8%) into 18-nucleotide deletion, and four patients (15.4%) into other insertion/substitution variant subgroups. The patient subgroup with 15-nucleotide deletion showed significantly longer progression-free survival than patients in other mixed insertion/substitution variant subgroup (*p* = 0.0244).

**Conclusions:**

The clinical significance of molecular variants of exon 19 deletion on the first line afatinib monotherapy is reported here for the first time. Further investigation is needed for development of better therapeutic strategies.

**Trial registration:**

This trial was registered at UMIN Clinical Trials Registry at 2014/12/4 (UMIN000015847).

## Background

Epidermal growth factor receptor (*EGFR*) tyrosine kinase inhibitors (TKIs) have been standard provision for advanced non-small cell lung cancer (NSCLC). Afatinib, a second generation *EGFR*-TKI, is an irreversible inhibitor that targets the EGFR and other members of the ERBB tyrosine kinase receptor family. It allows longer progression-free survival (PFS) in patients with NSCLC harboring *EGFR*-sensitizing mutations compared with platinum based chemotherapy [1, 2]. It has been widely approved in many countries as the first line treatment for NSCLC tumors with *EGFR*-sensitizing mutations. Deletion mutations of exon 19 and the single point mutation exon 21 Leu858Arg (L858R) are the most common mutations of *EGFR*, their incidences in Japanese population are 48.2 and 42.7%, respectively [3]. While *EGFR*-TKIs have high binding affinity for these common mutations, they showed different sensitivity to *EGFR*-TKIs. Although first and second generation *EGFR*-TKIs have shown better survival outcomes in patients with exon 19 deletion than in patients with L858R in some studies, it remains inconclusive [1, 3–8]. Furthermore, exon 19 deletion mutation has several molecular variants including in-frame deletions, substitutions, and insertions [9, 10]. These variants are heterogeneous, and many deletions almost bind the amino acid residues leucine-747 (L747) to glutamic acid-749 (E749) (LRE) which is located at the *N*-terminus of the EGFR kinase domain C-helix. This is a key structure for function and activation of EGFR [11–13]. Minor variants that do not include these codons are also reported [9]. These molecular mutational variants of exon 19 deletion are associated with different clinical outcomes and might have predictive roles for TKIs [10, 13–17].

We conducted a prospective, multi-institutional phase II biomarker study of *EGFR* mutated, advanced NSCLC patients treated with afatinib (West Japan Oncology Group [WJOG] 8114LTR). Clinical outcomes were overall response rate 78.6% and median PFS 14.2 months [18, 19]. Additionally, presence of mutated *EGFR* in plasma cell-free DNA (cfDNA) and its alteration during treatment was suggested to be a good prognostic marker of afatinib [18, 19]. In this preplanned subset study, we identified the molecular variants of *EGFR* exon 19 deletion in the subset harboring exon 19 deletion of WJOG8114LTR and analyzed their correlations with clinical outcomes of treatment with afatinib.

## Methods

### Study design and patient population

We recruited pathologically confirmed and previously untreated recurrence or advanced (Stage IIIB/IV) NSCLC patients with *EGFR*-sensitizing mutations confirmed by approved commercial tests. They received afatinib monotherapy (40 mg q.d.) until progressive disease or unacceptable toxicity. Assessment of tumor response was based on response evaluation criteria in solid tumors (RECIST), version 1.1 [20]. The primary endpoint was concordance rate of *EGFR* mutation status between tumor tissue and plasma. Secondary endpoints included biomarker analyses, detection of mutated *EGFR* cfDNA using digital polymerase chain reaction (PCR) method, and identification of exon 19 deletion molecular variants in tumor tissue. This study was conducted in accordance with the Declaration of Helsinki and good clinical practice. The study protocol was approved by the ethics committees of all participating centers and written informed consent was obtained from all patients. This study was registered at Clinical Trials Registry UMIN (ID: 000015847).

### DNA extraction from tissue and blood samples

Genome DNA extraction from formalin-fixed, paraffin-embedded (FFPE) specimens of surgically resected tissue was performed in an independent clinical laboratory (SRL, Tokyo, Japan). Genomic DNA mass was measured using a spectrophotometer (NanoDrop 2000C; Thermo Fisher Scientific, Wilmington, DE) as per the manufacturer’s recommendation.

Peripheral whole blood collected in EDTA tubes (BD Vacutainer Systems, Franklin Lakes, NJ) was centrifuged at 1500×g for 10 min at 4 °C and the plasma supernatant was transferred to 50 mL conical tubes (BD Falcon, Corning, NY) and stored at − 80 °C until use. Plasma DNA was isolated using the QIAmp Circulating Nucleic Acid Kit (Qiagen, Hilden, Germany) according to the manufacturer’s protocol. DNA was eluted in AVE buffer (50 μL). Approximately 40 μL of plasma DNA was concentrated to about 10 μL by SpeedVac (Thermo Fisher Scientific, Waltham, MA). DNA concentration was measured by Qubit 2.0 Fluorometer (Life Technologies, Carlsbad, CA).

### Variant analysis of *EGFR* exon 19 deletion mutation

These molecular variants were the first twenty of the catalogue of somatic mutations in cancer (COSMIC) frequencies as listed in Additional file 1: Table S1 [21]. Deletion in exon 19 was analyzed by the blocking-oligo-dependent (bo) PCR and Luminex technology using the tumor tissues before afatinib treatment. By blocking non-target amplification, only target sequences are amplified using universal PCR primers [22, 23]. When template DNA is wild type, blocking oligo binds to the complementary locus, and primer extension is inhibited. When template DNA is mutated, blocking oligo cannot bind to the target so primer extension is prolonged. The blocker can be a modified DNA oligonucleotide that does not prime amplification, or a clamping probe. Using serial dilutions of mutant DNA, rhPCR had a high detection capability limit of boPCR was as low as 0.1% mutant copies (deletion in exon 19) in a background of wild-type copies. The following primer sequences were used: Forward, /5Biosg/CTC TCT CTG TCA TAG GGA CTC TGG ATC and reverse, /5Biosg/CAT GGA CCC CCA CAC AGC AAA G. For the amplification, the 25 μL reaction-solution contained 0.63 unit of Taq DNA Polymerase and Uracil DNA Glycosylase, 20 μL of 1.25 × boPCR Buffer (Final 2.5 mM Mg2+), 0.2 mM of each dNTP and 0.6 mM dUTP, 0.1 to 0.2 μM of primers, and blocking oligo were used. The PCR were performed on GeneAmp9700 system (Applied Biosystems/Life Technologies) with followed parameter: UDG reaction at 40 °C for 10 min and primary denaturation at 95 °C for 5 min, followed by 55 cycles at 95 °C for 10 s and at 60 °C for 30 s, and 94 °C for 10 min, finally holding at 72 °C.

### Detection of *EGFR* mutation using plasma DNA

Plasma DNA was obtained from patients at baseline, weeks 2, 4, 8, 12, 24, 48, and at disease progression. Three types of clinically relevant *EGFR* mutations (exon 19 deletion, exon 20 Thr790Met [T790 M] and exon 21 L858R) were analyzed using plasma cfDNA with multiplexed, pico-droplet digital PCR assay (RainDrop system, RainDance Technologies, Billerica, MA) [24, 25]. Positivity of cfDNA was defined as mutant allele event/frequency of exon 19 deletion, exon 21 L858R, or exon 20 T790 M above the cutoff by digital PCR in plasma. Plasma mutant allele frequency (MAF) was calculated by dividing the number of copies of the mutant alleles by the total number of copies of the alleles at the specific locus.

### Statistical analysis

PFS was estimated by the Kaplan-Meier method with log-rank tests. Proportional hazards model was used for multivariate analysis. A *p*-value < 0.05 was regarded as statistically significant. Data were analyzed using JMP 13 software (SAS Institute Inc., Cary, NC, USA).

## Results

### Patient characteristics

**Fig. 1:**
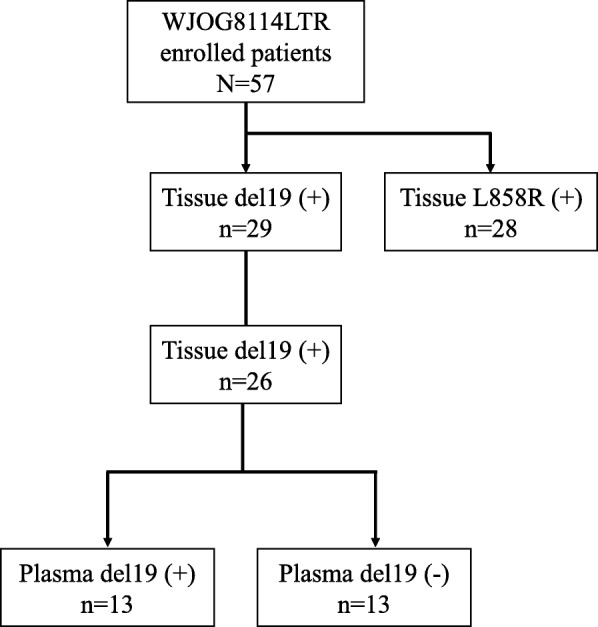
Consort diagram. Three patients who had exon 19 deletion were excluded (Rapid progression after enrollment (*n* = 1), Patients’ tumor DNA were unavailable (*n* = 2)). Del19: exon 19 deletion; L858R: exon 21 Leu858Arg

Fifty-seven patients were registered in the parental WJOG 8114LTR study. All patients had *EGFR*-sensitizing mutations, and 29 (50.9%) and 28 (49.1%) patients had exon 19 deletion and L858R, respectively (Fig. 1). All patient backgrounds and patients with tissue exon 19 deletion are shown in Table 1. Among patients with tissue exon 19 deletion, sixteen (55.2%) and 13 patients (44.8%) were male/female, respectively. Fifteen patients (51.7%) had stage IV disease, and seventeen patients (58.6%) had history of smoking. Performance status (PS) of all patients were either 0 or 1. Concerning exon 19 deletion subgroup, tissue specimens and/or blood samples of three patients were unavailable because of rapid progression before administration of afatinib and tissue insufficiency. We then analyzed data of 26 patients whose tumor tissue and plasma cfDNA were available in this study. Of these patients, thirteen (50.0%) were positive for plasma mutated *EGFR* at baseline blood draw (Fig. 1). These exon 19 deletion tumors included seven different variants (Table 2). The most common was p.E746_A750delELREA (*n* = 13, 50.0%), followed by different nucleotide variant of p.E746_A750delELREA (*n* = 4, 15.4%), p.L747_T751delLREAT (n = 4, 15.4%), p.E746_S752 > V (*n* = 2, 7.7%). All but one bound the amino acid residues L747 to E749 (LRE), 22 patients (84.6%) and 1 patient (3.8%) had short in-frame deletions (15–18 nucleotides) and macrodeletion (more than 20 nucleotides), respectively. This incidence and distribution was similar to those of COSMIC database (Table 2, Additional file 1: Table S1) [21].

**Table 1:** Background of all patients and patients with exon 19 deletion

		All patients (*N* = 57)	tissue del19^a^ (*n* = 29)
Gender	Male	26 (45.6%)	16 (55.2%)
	Female	31 (54.4%)	13 (44.8%)
Age	Median (Range)	69 (37–78)	68 (37–77)
Stage	Postoperative recurrence	16 (28.1%)	13 (44.8%)
	III	2 (3.5%)	1 (3.4%)
	IV	39 (68.4%)	15 (51.7%)
Smoking history	Yes	21 (36.8%)	17 (58.6%)
	No	36 (63.2%)	12 (41.4%)
Performance status	0	24 (42.1%)	8 (27.6%)
	1	33 (57.9%)	21 (72.4%)

^a^del19, exon 19 deletion

**Table 2:** Molecular variant distribution of *EGFR* exon 19 deletion mutation

Type of mutation (Amino Acid)	Type of mutation (Nucleotide)	Number of nucleotide deletion	Frequency (n = 26)	COSMIC^a^ ID	Number of COSMIC registered samples^b^
p.E746_A750delELREA	c.2235_2249del15 (Deletion)	15	13 (50.0%)	COSM6223	1106
p.E746_A750delELREA	c.2236_2250del15 (Deletion)	15	4 (15.4%)	COSM6225	528
p.E746_S752 > V	c.2237–2255 > T (complex)	Mixed ins/sub^c^	2 (7.7%)	COSM12384	70
p.E746_T751 > I	c. 2235–2252 > AAT (complex)	Mixed ins/sub	1 (3.8%)	COSM13551	4
p.L747_T751delLREAT	c.2240_2254del15 (Deletion)	15	4 (15.4%)	COSM12369	134
p.L747_P753 > S	c.2240–2257 del18 (Deletion)	18	1 (3.8%)	COSM12370	174
p.S752_I759delSPKANKEI	c.2253–2276 del24 (Deletion)	24	1 (3.8%)	COSM13556	9

^a^the Catalogue Of Somatic Mutations In Cancer, ^b^At 07/11/2018, ^c^Mixed insertion/substitution

### Subgroup classification of exon 19 deletion molecular variants

Based on previous studies, exon 19 deletion variants were categorized according to several classifications based on the number of deleted nucleotides, on deletion starting codons, and the common in frame deletion [10, 14–17]. With the classification based on the number of deleted nucleotides, p.E746_A750delELREA and p.L747_T751delLREAT were classified as 15-nucleotide deletion subgroup (15n-del), p.L747_P753 > S was classified as 18-nucleotide deletion subgroup (18n-del) and p.E746_S752 > V and p.E746_T751 > I were classified other mixed insertion/substitution variant subgroup (other/mixed ins/sub) based on the mutation syntax of COSMIC database [21]. p.S752_I759delSPKANKEI, which showed 24 nucleotide deletion (macrodeletion) was also classified to other/mixed ins/sub subgroup. Ultimately, twenty-one patients (80.8%) were classified into 15n-del, one patient (3.8%) into 18n-del, and four patients (15.4%) into other/mixed ins/sub subgroups (Table 3). Concerning deletion starting codon classification, twenty (76.9%) and five patients (19.2%) whose deletion starting codon was E746 and L747 were classified into E746 and L747 subgroups, respectively. E746 and L747 subgroups included the amino acid residues LRE, therefore 25 patients (96.2%) were classified into LRE and one patient (3.8%) into non-LRE subgroup (Table 3). On the other hand, all 15n-del and 18n-del belonged to the starting codon E746 or E747 subgroups (Table 2). Similar to COSMIC report, the most frequently detected exon 19 deletion variant was p.E746_A750delELREA and 17 patients (65.4%) were classified into “ELREA” subgroup (Table 3).

**Table 3:** Patient distributions according to exon 19 deletion molecular subtype classifications

		All del19 patients (*n* = 26)
Number of nucleotide deletion	15-nucleotide deletion (15n-del)	21 (80.8%)
	18-nucleotide deletion (18n-del)	1 (3.8%)
	Other insertion/substitution (other/mixed ins/sub)	4 (15.4%)
Deletion starting codon	E746 group	20 (76.9%)
	L747 group	5 (19.2%)
	Non-LRE group	1 (3.8%)
LRE or non-LRE	LRE group	25 (96.2%)
	non-LRE group	1 (3.8%)
ELREA or not	ELREA group	17 (65.4%)
	non-ELREA group	9 (34.6%)

### Association of EGFR exon 19 subtypes and clinical outcome

**Fig. 2:**
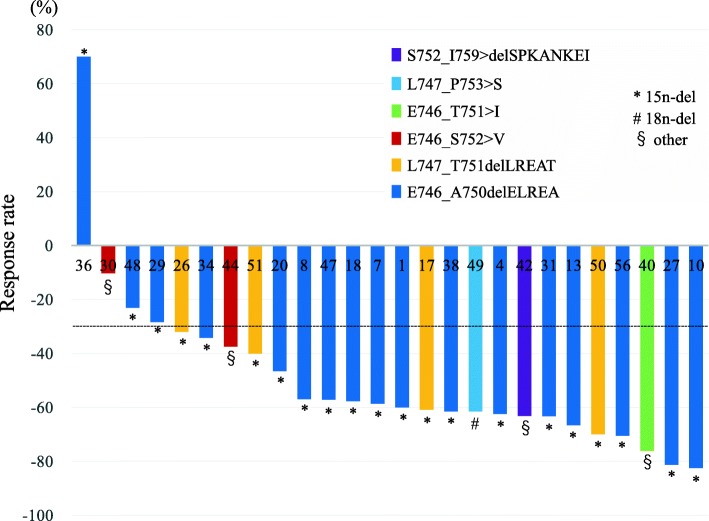
Waterfall plots of overall response rate according to different exon 19 deletion molecular variants (*n* = 26). The numbers on the X-axis are case numbers. A dashed line means 30% decline of tumor volume; partial response. 15n-del: 15-nucleotide deletion subgroup; 18n-del: 18-nucleotide deletion subgroup; other: other/ mixed insertion/substitution variant subgroup

**Fig. 3:**
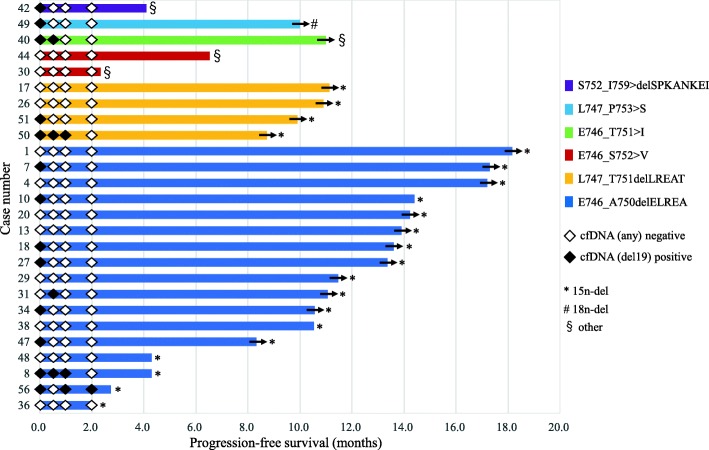
Swimmer plots of Progression-free survival according to different exon 19 deletion molecular variants (n = 26). Arrows on the right end of the bars indicate ongoing response. Quadrangles on each bar represent plasma cfDNA collection. Black-fill and blank represent positive and negative for plasma cfDNA (exon 19 deletion)

**Fig. 4:**
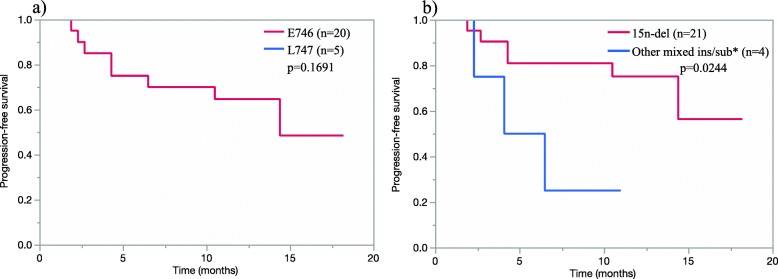
Progression-free survival for patients with exon 19 deletion according to different subtype classifications (*n* = 25). **a** Classification with deletion starting codon (*n* = 25). A patient with the starting codon S752 was excluded because of only one patient was in this subgroup. Any patients with the starting codon L747 did not experience disease progression at the data cut-off date. **b** Classification with the number of deleted nucleotides (*n* = 25). Other/mixed insertion/substitution subgroup includes microdeletion (24n-del) and insertion/substitution. A patient with 18 nucleotide deletion was excluded because of only one patient was in this subgroup. *Other/mixed ins/sub: other/mixed insertion/substitution subgroup

At the time of data cutoff, the overall response rate (RR) to afatinib monotherapy was 84.6% of the patients with exon 19 deletion. As shown in Fig. 2, each molecular variant of exon 19 deletions showed good response. As already reported, median PFS was 14.2 months in the parental WJOG 8114LTR study [19]. Subgroup analyses of PFS according to different classifications were then performed. Shown in the swimmer plot, 12 patients (60.0%) with the starting codon L746 and five patients (100.0%) with L747 were still on afatinib treatment, respectively (Fig. 3). Their Kaplan-Meier curves showed no difference of PFS (*p* = 0.1691) (Fig. 4a). On the other hand, 15 patients (71.4%) with 15 nucleotide deletion and one patient (25.0%) with other nucleotide deletion (other/mixed ins/sub) were still on the treatment and 15n-del subgroup showed significantly longer PFS than other/mixed ins/sub subgroup (*p* = 0.0244). The number of the patients who had p.E746_S752 > V, p.S752_I759 > del SPKANKEI was small, however, they tended to show shorter PFS, and it led to shorter PFS in other/mixed ins/sub subgroup (Figs. 3 and 4b). PFS of “ELREA” subgroup was similar to non-ELREA (*p* = 0.7442) (Additional file 2: Figure S1). As mentioned before, plasma cfDNA mutation positivity and its alteration during afatinib were suggested to be good prognostic markers of afatinib in the parental study WJOG 8114LTR [18, 19]. In this exon 19 deletion cohort, at baseline thirteen patients (50.0%) were positive for mutated *EGFR* cfDNA, and three patients (11.5%) were positive at four weeks. The swimmer plot suggested that the cfDNA negative patients at baseline and the patients whose baseline positive cfDNA subsequently turned negative tended to have longer PFS (Fig. 3). However, presence of cfDNA at baseline as well as at four weeks did not show statistically significant effects on PFS (*p* = 0.7452, *p* = 0.4609, respectively) (Additional file 3: Figure S2a, b). Concerning plasma MAF, no clear trend was found in the association between plasma MAF and treatment efficacy. Some patients with higher MAF (more than 10%) before treatment (patient #8: 91.4%, patient #40: 31.0%, patient #47: 32.8%, patient #50: 34.0%), and all but one showed longer PFS (Fig. 3). PFS of the patients with lower MAF, meanwhile, was inconsistent. Multivariate analysis which took into account these factors did not show any factors to predict better prognosis on afatinib monotherapy (Additional file 1: Table S2).

## Discussion

Exon 19 deletion and L858R point mutation activate somatic mutations in *EGFR*, and they frequently contribute to structural changes of EGFR tyrosine kinase domains which might be responsible for their different sensitivities to *EGFR*-TKIs [12]. Mentioned above, exon 19 deletion consists of several molecular variants. In this study, we evaluated the impact of exon 19 deletion molecular variants on sensitivity to first line afatinib monotherapy.

According to COSMIC database, the most frequent molecular variant was p.E746_A750delELREA, followed by p.L747_P753 > S, p.L747_T751delLREAT and p.L747_A750 > P (Additional file 1: Table S1) [21]. While these variants almost always include LRE area, other variants aside from LRE, such as p.S752_I759delSPKANKEI, were also identified [21]. The tyrosine kinase domain of EGFR consists of the N-lobe and the C-lobe. The frame-shift alterations of exon 19 deletion mutations often happen near the C-helix within the N-lobe because of its fragility. Correct positioning of C-helix is required for proper tyrosine kinase activation, and these frame-shift alterations contribute to conformational changes of the tyrosine kinase domain. Each molecular variant appears to have provided specific structural or surface potential changes of EGFR, this might result in variant specific downstream signaling activities [12, 13, 16, 26–28]. Ogasawara et al. recently reported that a single codon deletion E746del could induce transformation of the C-terminal domain of EGFR in 3-D structure analysis. The mutant was more kinetically activated and possessed altered electrostatic surface potential of gefitinib binding sites, which preceded much more potent inhibition by gefitinib than wild-type *EGFR*, and its interaction with gefitinib was similar to common deletion, such as p.E746_A750delELREA [28]. Improta et al. showed that uncommon exon 19 deletion mutations, such as complex deletions with insertion, macrodeletions and duplications also had important structural changes involving the C-helix. Such mutations did not have the same sensitivities to *EGFR*-TKI [13]. Hence, a single codon deletion as well as complex deletion and microdeletion harbor their own structural conformations which might introduce different sensitivities to *EGFR*-TKIs [12, 13, 16, 26–28]. Even though it remains hypothetical owing to the small number of events, afatinib is an irreversible *EGFR*-TKI which has broader and more durable inhibitory potencies than those of the first generation *EGFR*-TKIs, such as gefitinib. In our present study, some exon 19 deletion molecular variants that have specific structural changes of EGFR may provide different impacts on the efficacy of irreversible *EGFR*-TKI. This could result in different sensitivities between patients harboring different exon 19 variants.

Several reports discuss the similar influences of exon 19 deletion variants on the patients with various stages and treatment lines of different *EGFR*-TKIs to date (Additional file 1: Table S3) [10, 14–17]. These reports employed several classifications including by the deletion starting codon and by the number of deleted nucleotides. Concerning the starting codon classification, Lee et al. and Kaneda et al. concluded that E746 had longer PFS than E747, although other reports did not [10, 14–17]. Regarding the number of deleted codons, only Lee et al. reported that 15n-del revealed longer PFS than 18n-del. They also reported that complex deletions with insertion or substitution had much better PFS, but Kaneda et al. stated the opposite conclusion [15, 16]. In our report, according to classification by the number of deleted codons, 15n-del showed longer PFS than complex and 24n-del. This result was contrary to the results of Lee et al. but consistent with the results of Kaneda et al. [15, 16] Despite the preferable response of 15n-del to afatinib in the present study, some patients with 15n-del still showed poor response. Similar phenomenon was reported in the patients that had *EGFR*-TKI-sensitizing mutations in previous studies [29]. Co-occurring mutations may confer poor response to afanitib monotherapy despite the presence of 15n-del. Additional comprehensive biomarker analysis may help to explore mechanisms of resistance to afatinib, e.g. *MET* amplification, *BRAF* V600E, and small cell transformation [ 30].

Previous reports and parental analysis of this study conversely suggest that detection of plasma-mutated DNA may be a good prognostic marker candidate [19, 31, 32]. With our comparatively small subset, however, similar prognostic impacts were not obtained. Furthermore, concerning plasma MAF, there was no clear correlation between plasma MAF and treatment efficacy.

To our knowledge, this is the first report that collected tissue samples to detect exon 19 deletion molecular variants centrally and to evaluate their influence on the first line afatinib monotherapy. The small sample size is a considerable limitation of this study, so further data accumulation and detailed analyses of the molecular variants are still needed to more fully understand the actual difference of sensitivity to *EGFR*-TKIs. It would be greatly advantageous for upcoming personalized medicine.

## Conclusion

Understanding differential prognosis of the molecular variants of exon 19 deletion might lead us to select more appropriate treatment using *EGFR*-TKIs that allow better patient outcomes.

## Supplementary information

**Additional file 1: Table S1.** Exon 19 deletion variant list. These molecular variants were the first twenty of the Catalogue Of Somatic Mutations In Cancer (COSMIC) frequencies. ^#^At 07/11/2018. **Table S2.** Multivariate analysis of progression-free survival with the patients with exon 19 deletions receiving afatinib (*n* = 24). A patient with the starting codon S752 and another patient with 18 nucleotide deletion were excluded because only one patient was in each subgroup. **Table S3.** Previous reports of non–small cell lung cancers with different exon 19 deletions molecular variants and their clinical outcomes. *referred to 15n-del (ELREA).

**Additional file 2: Figure S1.** Progression-free survival for patients according to different subtypes of exon 19 deletions (*n* = 26). ELREA or not.

**Additional file 3: Figure S2.**Progression-free survival for patients with exon 19 deletion according to presence of mutated plasma *EGFR*. a) At baseline (n = 26), b) At 4 weeks (n = 26). del19, exon 19 deletion.

## Data Availability

The datasets generated and/or analysed during the current study are not publicly available due patients right of confidentiality but are available from the corresponding author on reasonable request.

## Abbreviations

Del19: Exon 19 deletion
EGFR: Epidermal growth factor receptor
ELREA: Glutamic acid-746 (E746) to alanine-750 (A750)
LRE: Leucine-747 (L747) to glutamic acid-749 (E749)
NSCLC: Non-small cell lung cancer
PCR: Polymerase chain reaction
PFS: Progression-free survival
RR: Response rate
TKI: Tyrosine kinase inhibitors
