# Supplementary material for: Differential significance of molecular subtypes which were classified into EGFR exon 19 deletion on the first line afatinib monotherapy
Source: BMC Cancer. 2020 Feb 6;20:103. doi: 10.1186/s12885-020-6593-1 (PMC7006223; doi:10.1186/s12885-020-6593-1)
Supplement: Supplementary file 1 — Additional file 1: Table S1. Exon 19 deletion variant list. These molecular variants were the first twenty of the Catalogue Of Somatic Mutations In Cancer (COSMIC) frequencies. #At 07/11/2018. Table S2. Multivariate analysis of progression-free survival with the patients with exon 19 deletions receiving afatinib (n = 24). A patient with the starting codon S752 and another patient with 18 nucleotide deletion were excluded because only one patient was in each subgroup. Table S3. Previous reports of non–small cell lung cancers with different exon 19 deletions molecular variants and their clinical outcomes. *referred to 15n-del (ELREA). [file 12885_2020_6593_MOESM1_ESM.docx]

Additional Table S1. Exon 19 deletion variant list.

| Amino Acid mutation (COSMIC ID) | Number of COSMIC registered samples^#^ | Amino Acid mutation (COSMIC ID) | Number of COSMIC registered samples |
| --- | --- | --- | --- |
| p.E746_A750delELREA (COSM6223) | 1106 | p.S752_I759delSPKANKEI (COSM13556) | 9 |
| p.E746_A750delELREA (COSM6225) | 528 | p.S752_I759delSPKANKEI (COSM6256) | 8 |
| p.L747_P753>S (COSM12370) | 174 | p.L747_T751>S (COSM6210) | 7 |
| p.L747_T751delLREAT (COSM12369/6254) | 134 | p.L747_P753>Q (COSM12387) | 7 |
| p.L747_A750>P (COSM12382) | 98 | p.L747_A750>P (COSM12422) | 7 |
| p.E746_S752>V (COSM12384) | 70 | p.E746_S752>D (COSM6220) | 5 |
| p.L746_T751>A (COSM12678) | 46 | p.E746_T751delELREAT (COSM12728) | 5 |
| p.L747_S752delLREATS (COSM6255) | 41 | p.E746_S752>A (COSM12367) | 4 |
| p.L747_T751>P (COSM12383) | 27 | p.E746_T751>I (COSM13551) | 4 |
| p.L747_E749delLRE (COSM6218) | 15 | p.L747_T751>Q (COSM12419) | 2 |

Additional Table S2

|  | Hazard ratio | 95% CI | P-value |
| --- | --- | --- | --- |
| Grouping; starting codon  E746 vs L747 | 9.479x10^-10^ | 0.0-1.2385 | 0.0713 |
| Grouping; number of deleted nucleotide  Other mixed insertion/substitution vs 15n-del | 3.6755 | 0.4982-19.7639 | 0.1767 |
| Baseline plasma cfDNA  Positive vs Negative | 0.4512 | 0.0641-2.1130 | 0.3224 |
| 4w plasma cfDNA  Positive vs Negative | 9.8462 | 0.4106-134.9310 | 0.1319 |

Additional Table S3. Previous reports of non–small cell lung cancers with different exon 19 deletions molecular variants and their clinical outcomes.

|  |  | PFS (months) | p value | OS (months) | p value |
| --- | --- | --- | --- | --- | --- |
| Chung et al (14).  gefitinib or erlotinib | E746 | 9.8 | 0.665 | 33.1 | 0.776 |
|  | L747 | 10.5 |  | 32.8 |  |
|  | non-LRE | 5.9 |  | 33.7 |  |
| Lee et al (15).  1^st^ line  gefitinib or erlotinib | 18n-del | 6.5 | 0.012 | 17.7 | 0.240 |
|  | 15n-del | 12.4 |  | 23.8 |  |
|  | insertion/substitution | 22.3 |  | not reached |  |
|  | E746 | 14.2 | 0.021 | 24.6 | 0.307 |
|  | L747 | 6.5 |  | 17.7 |  |
| Kaneda et al (16).  Any line  gefitinib or erlotinib | E746 | 11.7 | 0.022 | 47.4 | 0.855 |
|  | L747 | 10.0 |  | 31.5 |  |
|  | with insertion/substitution | 10.0 | 0.024 | 23.2 | 0.439 |
|  | without insertion/substitution | 11.7 |  | 47.4 |  |
| Su et al (17).  gefitinib or erlotinib | E746 | 11.6 | 0.463 | 22.5 | 0.464 |
|  | L747 | 14.1 |  | 26.8 |  |
|  | non-LRE | 16.0 |  | 27.7 |  |
| Sutiman et al (10).  1^st^ line  gefitinib | E746 | 10.9 | 0.781 | 20.0 | 0.738 |
|  | L747 | 10.5 |  | 19.0 |  |
|  | 15n-del (ELREA) | 11.1 | 0.082 | 19.9 | 0.066 |
|  | 15n-del (non-ELREA) | 6.87 |  | 11.3 |  |
|  | 18n-del | 9.53 | 0.645* | 19.0 | 0.813* |
|  | mix/del/ins/sub (del<15n) | 10.0 | 0.325* | 22.9 | 0.640* |
|  | mix/del/ins/sub (del>15n) | 17.4 | 0.199* | 27.8 | 0.015* |
