# Supplementary figures and images for: Differential significance of molecular subtypes which were classified into EGFR exon 19 deletion on the first line afatinib monotherapy
Source: BMC Cancer. 2020 Feb 6;20:103. doi: 10.1186/s12885-020-6593-1 (PMC7006223; doi:10.1186/s12885-020-6593-1)

## Slide 1
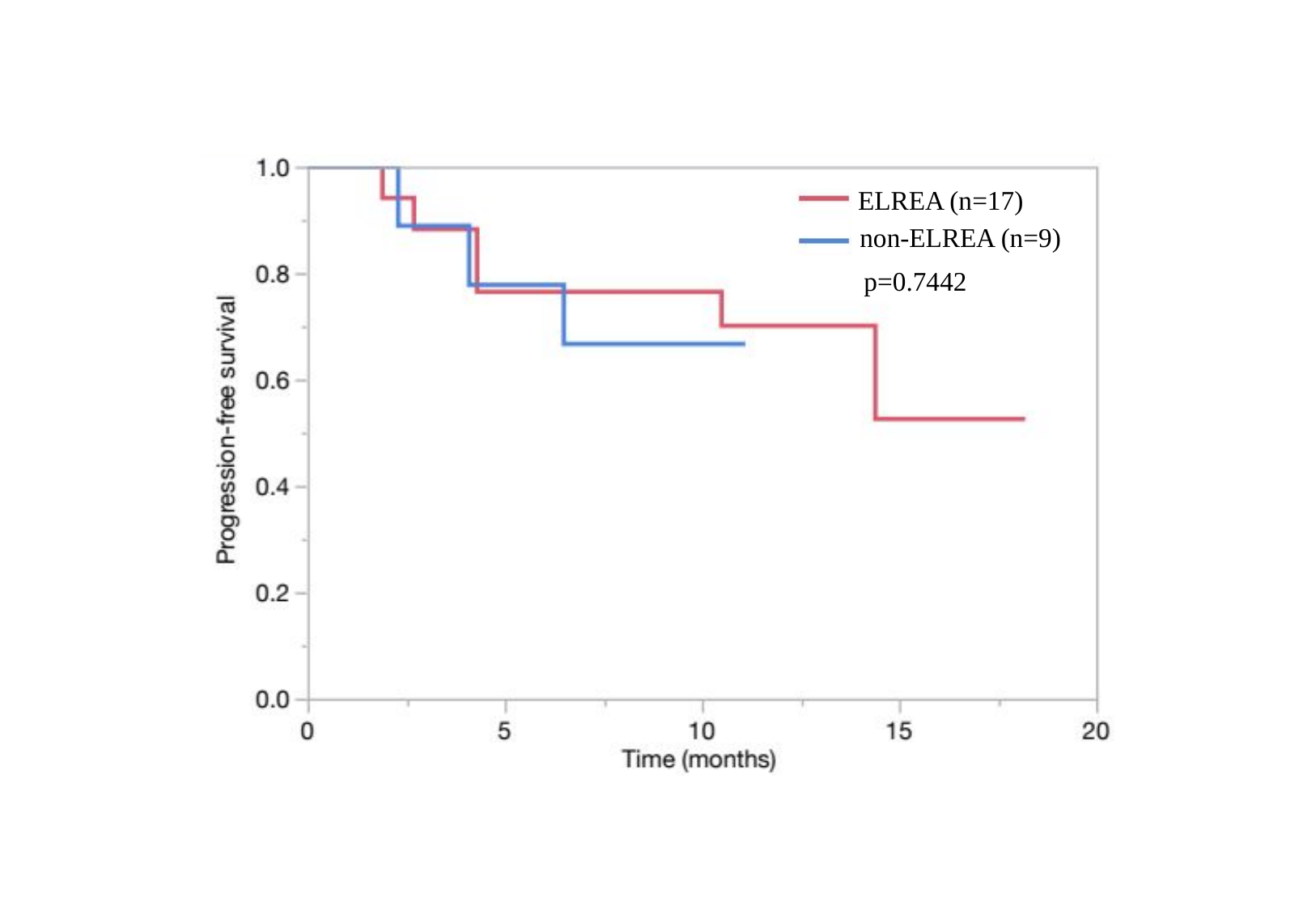

ELREA (n=17)
non-ELREA (n=9)
p=0.7442

Supplement: Supplementary file 2 — Additional file 2: Figure S1. Progression-free survival for patients according to different subtypes of exon 19 deletions (n = 26). ELREA or not. [file 12885_2020_6593_MOESM2_ESM.pptx]

## Slide 1
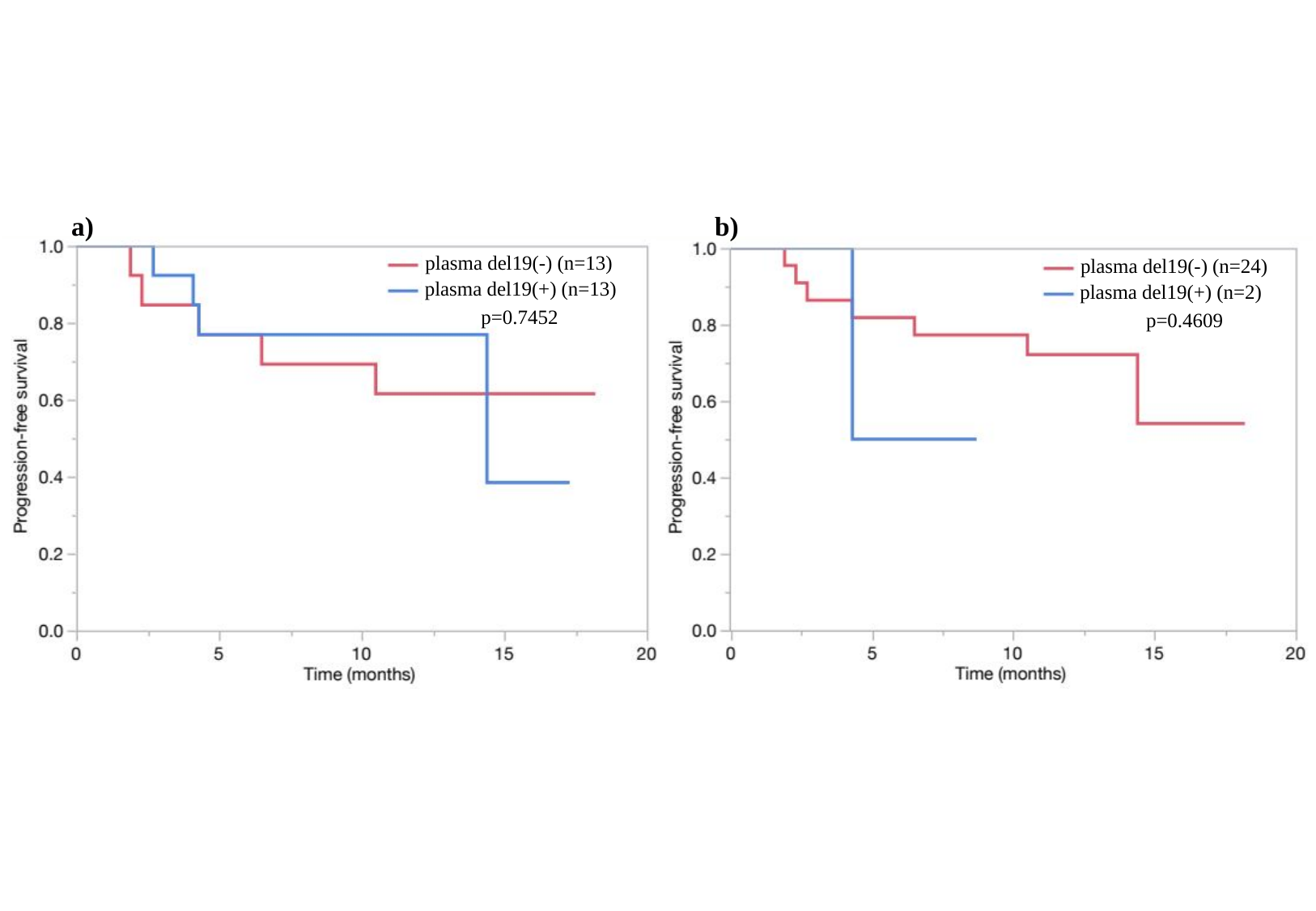

a)
plasma del19(-) (n=13)
plasma del19(+) (n=13)
p=0.7452
b)
plasma del19(-) (n=24)
plasma del19(+) (n=2)
p=0.4609

Supplement: Supplementary file 3 — Additional file 3: Figure S2.Progression-free survival for patients with exon 19 deletion according to presence of mutated plasma EGFR. a) At baseline (n = 26), b) At 4 weeks (n = 26). del19, exon 19 deletion. [file 12885_2020_6593_MOESM3_ESM.pptx]
